# 3D Finite Element Study of the Physiological Anchorage Control Concept on Anchorage Molars in Lingual Orthodontics

**DOI:** 10.1155/2022/1421586

**Published:** 2022-11-22

**Authors:** Jiayuan Zhao, Majing Su, Qian Zhao, Jiajie Liu, Junbin Wang, Junjie Wang, Xiaoli An

**Affiliations:** School/Hospital of Stomatology, Lanzhou University, Lanzhou 730000, China

## Abstract

**Objective:**

To study the effect of the physiological anchorage control concept on anchorage molars in lingual and labial orthodontic techniques.

**Methods:**

Three-dimensional finite element models, including the right maxillary first molar, periodontal ligament, alveolar bone, and buccal tube, were established. The models were divided into the McLaughlin–Bennett–Trevisi (MBT™) straight-wire model with 0-degree maxillary first molar axial inclination and the physiologic anchorage Speewire system (PASS) model with −7-degree maxillary first molar axial inclination. Simulated sliding retraction forces (1 N, 1.5 N, and 2 N) were loaded on the buccal side and lingual side, and retraction forces (0.5 N, 0.75 N, and 1 N) were loaded on the buccal and lingual sides simultaneously. The displacements, principal stresses, and von Mises stresses of the periodontal ligament under different conditions were derived.

**Results:**

The anchorage molars showed different degrees of rotation, tipping, intrusion, and extrusion. As the force increased, these displacement trends also increased. The mesial displacement of the buccal + lingual force loading was less than that of the other two groups. Under the same force load method, the mesial displacement of the PASS group was less than that of the MBT group. Tilt movement increases the tensile stress of the distal cervical margin and root mesial apical third and the compressive stress of the mesial cervical margin and root distal apical third. The maximum stress of the periodontal ligament was less than that of the other two groups when the lingual force was loaded.

**Conclusion:**

The physiological anchorage control concept in lingual orthodontics provides better sagittal anchorage control than in labial orthodontics, but there is no significant difference numerically. Attention should be given to the control of torsion, torque, and arch width. Tilt movement increases the PDL stress of the cervical margin and root apical third. The sliding retraction force should be loaded lingually to maintain the force value of 1∼1.5 N.

## 1. Introduction

Anchorage control is incorporated in every step of orthodontic treatment, and it can directly control the treatment effect [1], especially in patients with protrusion deformities treated with extraction, to enhance molar anchorage and various devices, such as facebow, transpalatal arch, and Nance holding arch, are used in the clinic. However, the application of these devices may depend on patient cooperation, cause discomfort and other problems, and produces limited effect [2, 3]. Miniscrew implants are considered to achieve maximum anchorage control [4], but the risks of fracture, expulsion, damage to adjacent tissues, and root and alveolar bone absorption have been reported [5–7], which are also expensive. Therefore, methods to safely, comfortably, simply, and effectively enhance anchorage are urgently needed.

Based on years of research, the concept of physiological anchorage control was first proposed by Professor Xu [8], and the physiological anchorage control appliance, also known as the physiological anchorage Speewire system (PASS), was developed. It mainly improves the use of brackets [9] and the selective bonding of brackets, utilizes the principle of differential moments of the teeth [10], maximizes the use of physiological anchorage, and reduces the loss of mechanical anchorage to strengthen the anchorage. The cross-buccal tube (XBT) of PASS for the upper first molar is specially designed, consisting of a −7-degree inclination prescription and a −25-degree auxiliary tube. In the first stage of anterior alignment, the initial NiTi wire should be inserted in the −25-degree auxiliary tube into the upper arch. Not emphasizing the leveling of Spee's curve, PASS maintains the protection of the physiological curve, which ensures that no matter in which stage, the upper molars can be subjected to the backward tilting moment, which places them in an “anchorage preparation” state. A randomized controlled trial reported by Chen et al. [11] showed that compared with the McLaughlin–Bennett–Trevisi (MBT™) straight-wire appliance, PASS without additional anchorage devices could attain good anchorage control by considering the dentoalveolar compensation of the anchor teeth.

Lingual appliances first appeared in the 1970s [12]. In recent years, with the rapid development of oral biomechanics, indirect bonding, new archwires, computer-aided design, and manufacturing technology (CAD/CAM), 3D scanning, and other technologies, emerging personalized lingual orthodontics have attracted increasing attention from orthodontists and patients because of their better aesthetics and comfort [13–15]. Compared with labial orthodontics, the bracket spacing is shortened, the rigidity of the archwire is enhanced, and it is more advantageous to control the overall movement of molars in the retraction stage [16]. However, similar to labial orthodontics, anchorage loss is difficult to avoid without additional control. Therefore, we considered applying physiological anchorage control to lingual orthodontics to strengthen the molar anchorage. The three-dimensional finite element method (3D FEM), a noninvasive virtual measurement method, has become the first choice for orthodontic biomechanical studies [17]. Through this study, we provide theoretical guidance for the effective clinical application of an orthodontic force loading strategy. The purpose of our study was to study the displacement pattern of the anchorage molar and the change in periodontal ligament (PDL) stress when the extraction space was closed by a lingual appliance with the PASS concept by 3D FEM.

## 2. Materials and Methods

### 2.1. Establishment of 3D FE Models

A healthy adult male volunteer with normal occlusion had a maxillary first molar without caries and obvious abrasion, and the periodontal tissue was healthy. After the volunteer provided informed consent and signed the informed consent form, cone-beam computed tomography (CBCT) was performed in the radiology department of the Hospital of Stomatology Lanzhou University. To obtain the data of the maxillary first molars more accurately, the volunteer was asked to bite an X-ray transmitted object to separate the upper and lower dentition when undergoing CBCT. The thickness of the CBCT layer was 0.25 mm, and the scanning time was 14.7 s. The acquired data were imported into Mimics Medical (Materialise, Flemish Brabant, Belgium) software in DICOM format. After defining the three-dimensional directions, the digital three-dimensional model of the right maxillary first molar was extracted according to the threshold of the tooth, and the data were saved in STL format and imported into Geomagic Studio (Raindrop Geomagic, North Carolina, USA) software for reverse reconstruction. The PDL model was established by extending the root outward by 0.25 mm. The tooth and PDL models were refined, the materialized model was generated by reverse reconstruction, and a simplified model simulating alveolar bone was generated at the periphery of the PDL.

According to the standard dimensions of the buccal tube of the MBT appliance (3B, Zhejiang, China) and the XBT (Shinye, Zhejiang, China), the 3D models of the MBT buccal tube, XBT, and lingual tube were established by SolidWorks (Dassault Systèmes SolidWorks Corporation, Massachusetts, USA) software, and the data were stored in STL format.

The tooth, PDL, alveolar bone, and buccal tubes were assembled in SolidWorks software. MBT and XBT buccal tubes were assembled in the clinical crown center in the buccal group. Similar to the clinical bonding position, a lingual tube was assembled in the gingival side of the clinical crown center to reduce occlusal interference. According to the data of the MBT buccal tube and XBT, the 3D model of the MBT group with 0-degree axis inclination and the PASS group with −7-degree axis inclination of the right maxillary first molar were established. All of the models were imported into ANSYS Workbench (Ansys, Pennsylvania, USA) software in X-T format for the subsequent experiments.

### 2.2. Material Properties Setting

Each material was defined as isotropic, and the mechanical parameters were set according to reference [18, 19] (Table 1).

The model was divided into tetrahedral elements. The solid model of teeth, periodontal ligament, and alveolar bone was divided into free meshes in ANSYS software, and the total numbers of nodes and elements were 29,060 and 15,707, respectively.

**Figure 1:**
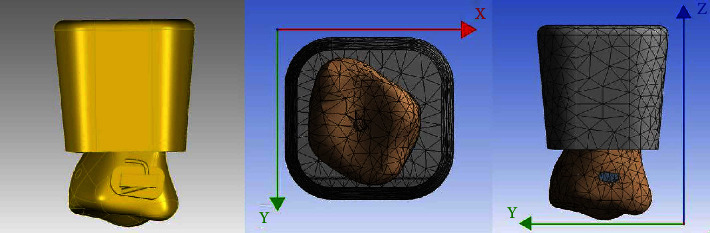
Established model and coordinate settings.

The degree of freedom of the alveolar bone was set as a complete constraint. The corresponding interaction between the buccal tube and teeth, teeth and PDL, PDL, and alveolar bone was set as bonded. The vertical direction was set as the *Z* axis, and the gingival direction was positive. The sagittal direction was set as the *Y* axis, and the distal direction was positive. The coronal direction was set as the *X* axis, and the lingual direction was positive (Figure 1).

### 2.3. Working Condition Settings

The 1 N, 1.5 N, and 2 N simulated sliding retraction forces (more precisely, they are the reacting force of the sliding retraction force) were loaded on the buccal side or lingual side, respectively, and 0.5 N, 0.75 N, and 1 N simulated sliding retraction forces were loaded on the buccal side and lingual side simultaneously (1 N, 1.5 N, and 2 N in the aggregate). The force in each working condition is parallel to the *Y*-axis and directing mesially. The specific settings of working Conditions 1∼18 are shown in Table 2.

Finally, all the above models were solved in the ANSYS Workbench software, and the results of the tooth displacement pattern, the first and third principal stresses, and the von Mises stresses of the PDL were derived and analyzed.

## 3. Results

### 3.1. Total Displacement Pattern

**Figure 2:**
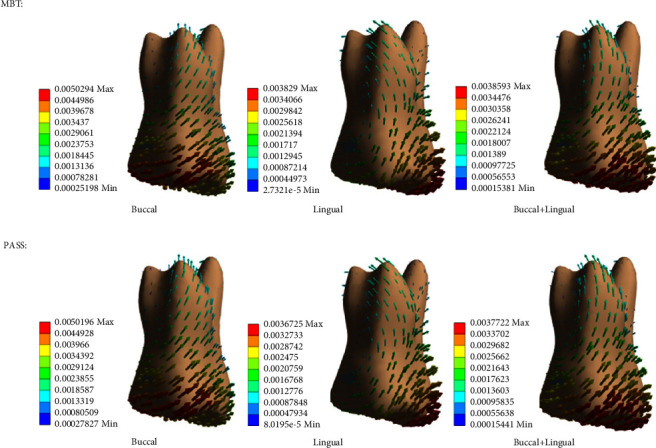
Displacement pattern vector analysis under 1.5 N retraction force.

In our study, the maxillary first molar, as an anchorage molar, after being loaded with the reaction force, the movement of the crown inclined mesially, and the apex inclined distally, which can be regarded as “anchorage loss” in the clinic. A slight buccal-lingual direction movement and a hardly observed intrusion movement were also found. Under the same force value, the maximum displacements of the buccal force and buccal + lingual force were very close, and both were less than the buccal force. The maximum displacement of the PASS group was less than that of the MBT group under the same force loading method. Vector analysis of the anchorage molar displacement pattern under 1.5 N retraction force is shown in Figure 2.

### 3.2. Displacement in Each Direction

**Figure 3:**
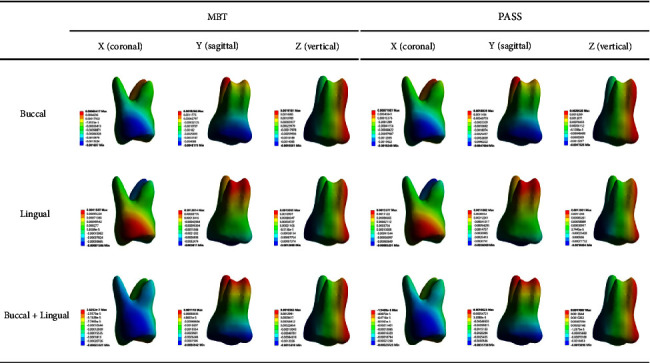
Displacement in each direction under 1.5 N retraction force (unit: mm).

**Figure 4:**
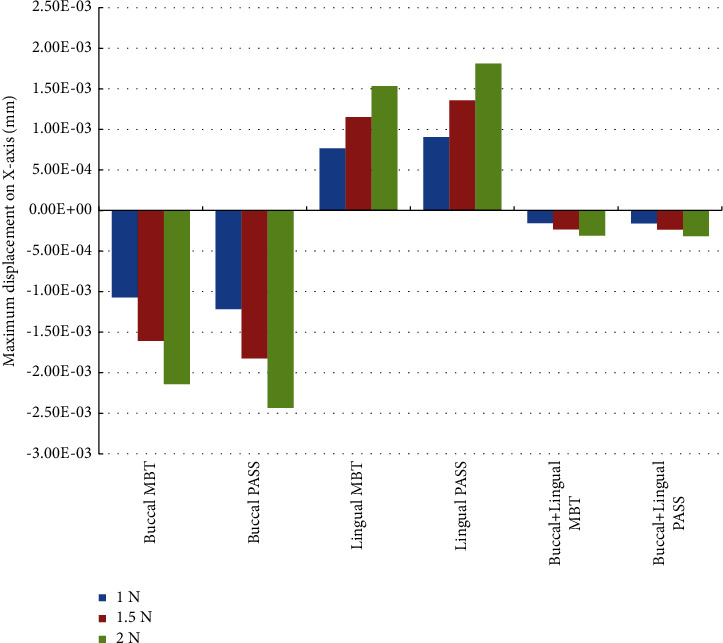
Maximum displacement on the *X*-axis in each working condition (unit: mm).

Under various force loading methods, all of the maximum displacements of the *X*-axis were observed on the lingual side of the crown distal surface, but the direction observed for buccal force and buccal + lingual force was negative (buccal direction), and the direction observed for lingual force was positive (lingual direction). Compared with the buccal and lingual forces, the distribution area of the maximum displacement was larger and more uniform under the buccal + lingual force (Figure 3). Under the same force loading value, the maximum displacement of the buccal + lingual force on the *X*-axis was significantly less than that of the other two force loading methods. Under the same force loading method, the buccal or lingual displacement in the PASS group was slightly greater than that in the MBT group (Figure 4).

**Figure 5:**
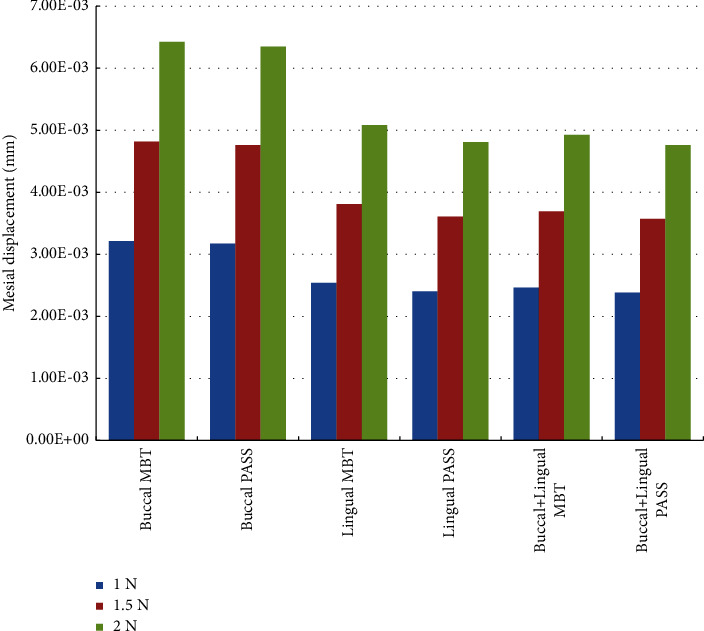
Maximum mesial displacement in each working condition (unit: mm).

All of the maximum displacements of the *Y*-axis were observed on the occlusal level of the crown, and the direction was negative (mesial direction), but the maximum displacement under the buccal force was distributed on the buccal side of the occlusal surface, the maximum displacement under the lingual force was distributed on the lingual side of the occlusal surface, and the maximum displacement under the buccal + lingual force was evenly distributed on the occlusal surface (Figure 3). Under the same force loading value, the maximum displacement under the buccal force on the *Y*-axis was greater than that of the other two force loading methods, and the maximum displacement values of the lingual force and buccal + lingual force were close. Under the same force loading method, the mesial displacement of the PASS group was less than that of the MBT group (Figure 5).

**Figure 6:**
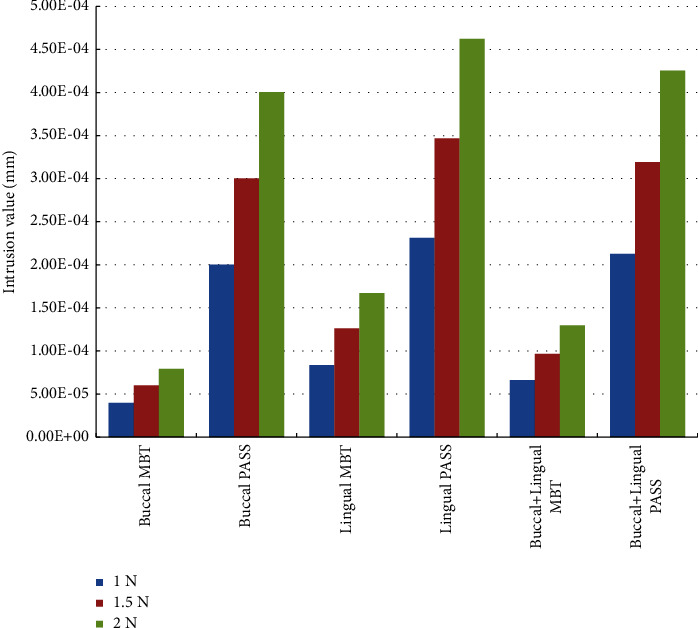
Intrusion value in each working condition. The result is obtained by calculating the sum of the maximum positive displacement and the maximum negative displacement (unit: mm).

For the *Z*-axis, the maximum negative (occlusal direction) displacement was observed on the lingual side of the crown distal surface, and the maximum positive (gingival direction) displacement was observed on the buccal side of the mesial surface of the crown and root (Figure 3). The values of the maximum positive displacement and the maximum negative displacement were very close, which indicates that almost all the displacements observed on the *Z*-axis came from the inclined movement of the teeth, but calculating their difference can be used to determine whether the teeth have a small amount of bodily extrusion or intrusion. In our study, according to our calculations, the maximum positive displacement value of the *Z*-axis under all force loading methods was greater than the maximum negative displacement value; therefore, we believe that there is a small amount of intrusion. It is worth noting that under the same force loading value, the intrusion of the lingual force loading is greater than that of the buccal and buccal + lingual forces; under the same force loading method, the intrusion of the PASS group was greater than that of the MBT group (Figure 6).

### 3.3. Stress the PDL

**Figure 7:**
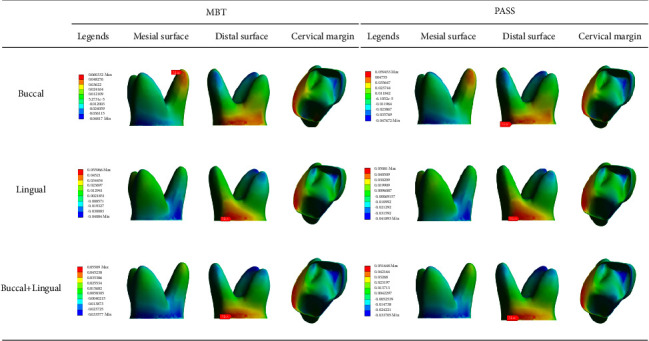
The first principal stress distribution in the PDL under 1.5 N force loading (unit: MPa).

**Figure 8:**
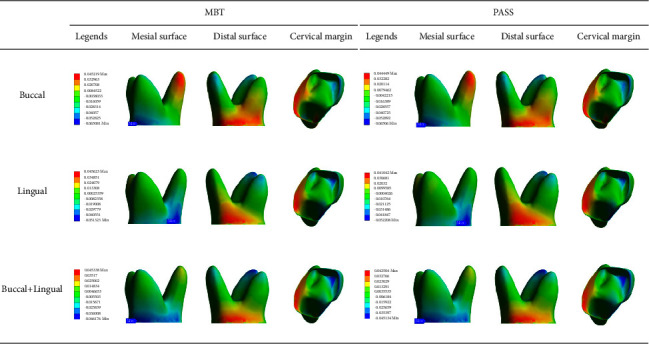
The third principal stress distribution in the PDL under 1.5 N force loading (unit: MPa).

The principal stress nephogram of the PDL under 1.5 N loading is shown in Figures 7 and 8. The first principal stress represents the tensile stress of the PDL. The tensile stress concentration was recorded on the mesial surface of the palatal root apical third and the cervical margin of the linguodistal surface in both the MBT and PASS groups when the buccal force was applied, but the maximum value in the MBT group was recorded on the mesial surface of the palatal root apical third, and the maximum value in the PASS group was recorded on the cervical margin of the linguodistal surface. When lingual and buccal + lingual forces were applied, the tensile stress concentration area of each group was in the cervical margin of the linguodistal surface, and no large tensile stress was observed in the apical third region. The third principal stress represents the compressive stress of the PDL. In all working conditions, compressive stress concentrations were observed in the cervical margin of the mesial surface, and no significant difference was observed between the MBT group and the PASS group. However, for different loading methods, buccal force is concentrated on the cervical margin of the buccomesial surface, lingual force application is concentrated on the cervical margin of the linguomesial surface, and buccal + lingual force is more evenly concentrated on the cervical margin of the mesial surface than the other two force loading methods. Notably, in addition to the mesial cervical margin, a large concentration of compressive stress was also observed at the root apical third of the mesiobuccal root, and it was most obvious when the buccal + lingual force was loaded.

Table 3 shows the maximum value of the first principal stress and the minimum value of the third principal stress of the PDL and the sites recorded in each working condition. It can be observed that with the increase in the force value, for the absolute value, the maximum values of the first principal stresses and the minimum values of the third principal stresses also increase. Under the same force value, compared with the absolute value, the minimum values of the third principal stresses under buccal force loading were greater than the maximum values of the first principal stresses, and the minimum values of the third principal stresses under lingual force and buccal + lingual force were less than the maximum values of the first principal stresses.

For von Mises equivalent stress, it can be observed that under the same force value, the maximum stress of the PASS group under buccal force loading is greater than that of the MBT group, the maximum stress of the PASS group under lingual force loading is less than that of the MBT group, and the maximum stress of the PASS group under buccal + lingual force loading is less than that of the MBT group, but the difference is not significant (Table 4).

## 4. Discussion

During the stage of extraction space closure, if an additional anchorage device was not applied, the first molar was the main anchorage. In mainstream straight wire technology, the Roth straight wire is used in the retraction spring method, while the Andrews and MBT straight wires are used in the sliding method [20]. The sliding method is also commonly used in PASS as it increases molar anchorage control through the improved XBT. At present, there is no unified conclusion on the specific value of the optimal sliding retraction force, which depends on the type of malocclusion, orthodontic technology, individual differences of patients, and the personal experience of doctors. In most studies and clinical applications, the range is 1∼2 N [21], and in some studies, researchers point out that the rate of tooth movement is positively correlated with the size of the orthodontic force within a certain range [22, 23]. However, Samuels et al. [24] reported that there was no significant difference in the rate of space closing between 1.5 N and 2 N retraction forces. In lingual orthodontics, the posterior has little impact on aesthetics; therefore, some doctors choose to apply retraction force on the buccal and lingual sides at the same time to reduce the occurrence of adverse effects such as molar torsion. In our study, to cover the common clinical situations as much as possible, 1 N, 1.5 N, and 2 N forces were simulated and loaded on the buccal side, lingual side, and buccal + lingual side simultaneously to provide a reference for the clinic. This is the first study on the application of the PASS concept in lingual orthodontics.

According to our results, compared with the other two methods, buccal + lingual force loading shows the least amount of mesial displacement, and buccal force loading shows the most mesial displacement, which suggests that buccal + lingual force loading has the best sagittal control of molars, and the sagittal control of molars under lingual force is better than buccal force.

The lingual orthodontic technique usually uses Andrews six elements as the terminal position when simulating tooth alignment [25], and the occlusal curve must be flat. However, compared with the MBT group, the concept of PASS is used to maintain the maxillary Spee's curve so that the molars are in a backward state. When retracting the anterior tooth, the anchorage molars have less mesial displacement. Our results show that PASS has fewer mesial displacement of the molar than MBT, but the difference is not significant from the numerical point of view, suggesting that we cannot rule out the possibility of using additional anchorage control even though PASS is used. For example, our results represent the combination of simultaneous buccal and lingual force loading and the concept of physiological anchorage control. However, considering the convenience of clinical operation and the comfort of patients, lingual PASS application may be a practical choice for clinical use because according to the results of this study, the lingual method is not significantly different from the buccal + lingual method (Figure 5). When a stronger anchorage is needed, miniscrew implant is still the trump card of orthodontists.

In all working conditions, the displacement pattern of the *Z*-axis (occlusal-gingival direction) is basically the same, manifested as mesial intrusion and distal extrusion, which is caused by mesial tipping of the anchorage molar. However, it can be observed that the maximum value of mesial intrusion is greater than the maximum value of distal extrusion, so we believe that there is a small amount of bodily intrusion of the anchorage molar. It should be noted that the intrusion of the PASS group was greater than that of the MBT group (Figure 6). The reason we speculate about this is that the angle between the retraction force line and the long axis of the tooth decreases, and the retraction force produces a greater intrusive component than that of the MBT group. However, a greater intrusive component may produce undesirable displacement in the horizontal direction (Figure 4). Especially when simply applying force on the buccal or lingual side, the force loading position is always on the buccal or lingual side of the center of resistance of the tooth, and then buccal or lingual inclination will inevitably occur. These results suggest that PASS may have a better vertical control effect, but since the order of magnitude of vertical displacement is much smaller than that of other directions, we cannot be definite of this conclusion. Attention should be given to the maintenance of the horizontal width. However, due to the limitations of the finite element method, these conclusions need to be further verified by clinical research.

In a 3D stress field, there are 3 principal stress components ranked in descending order [26]. We focus on the maximum value of the first principal stress and the minimum value of the third principal stress, which represent the maximum tensile stress and maximum compressive stress, respectively. As revealed by the results, tilt movement increases the tensile stress of the distal cervical margin and root mesial apical third and the compressive stress of the mesial cervical margin and root distal apical third. This is consistent with the work by Roscoe et al. [27]. High stress in the cervical margin indicates the risk of alveolar crest resorption, while high stress in the root apical third indicates the risk of root resorption. In some patients, the reduction of the alveolar crest and resorption of the apical root were found in the imaging examination after orthodontic treatment [28, 29].

There was no significant difference in the distribution of the PDL stress between the MBT group and the PASS group, but the change in the method of the force loading would significantly affect the distribution and value of the stress. Under the buccal + lingual force, the stress distribution in the cervical margin seemed to be more uniform than that of the other two types, but at the same time, a large stress concentration was observed at the apical third of the mesiobuccal root. From this point of view, simultaneous buccal and lingual force application is not necessarily the best choice.

Whether the principal stress or von Mises stress, under the same force value, when the retraction force is loaded buccally, the maximum stress of the PDL in the PASS group is greater than that in the MBT group, while when the retraction force is loaded lingually and buccally + lingually, the maximum stress of the PDL in the PASS group is less than that in the MBT group. We speculate that this may be because the change in the distance from the force loading position to the center of resistance (force arm) has a greater effect than that of the vertical component force on the PDL stress, but we still cannot explain this interesting result well.

According to the fourth strength theory, von Mises stress is used as an indicator of material failure. Lee [30] reported that the maximum stress that the periodontal ligament can sustain is 0.026 MPa, except lingual 1 N, buccal + lingual 1 N, and PASS lingual 1.5 N loading, the maximum values of other recorded stresses exceed 0.026. Therefore, it is recommended to load the retraction force on the lingual side and control the force value at 1∼1.5 N. However, due to the different PDL models used in all finite element studies, the safe range of PDL stress in finite element studies still needs to be further studied. Because we have adopted a linear periodontal membrane model, according to Roscoe et al. [27] the linear model will produce greater stress values than the nonlinear model under a load of heavy orthodontic force.

## 5. Limitation

First, due to the complexity of oral and maxillofacial systems and the limitations of computer technology, the finite element model is usually simplified. At present, researchers conducting most finite element studies analyze the tooth and periodontal tissue as isotropic linear materials, and the same is true of this study, thus reducing the authenticity of the results. Second, when the finite element method is used in the study of orthodontic biomechanics, it can only obtain the initial results under the application of orthodontic force, while the actual orthodontic tooth movement is affected by the absorption and reconstruction of alveolar bone, which is difficult to simulate by the finite element model, although the initial displacement trend may be consistent with the long-term tooth movement trend [31]. Third, this study is limited to the simulation of a single tooth. The presence of adjacent teeth and archwires may change the displacement of teeth to a certain extent, but we believe that the total movement pattern is similar.

## 6. Conclusions

The physiological anchorage control concept in lingual orthodontics has better sagittal anchorage control than in labial orthodontics, but there is no significant difference numerically. Attention should be given to the control of torsion, torque, and arch width. Tilt movement increases the PDL stress of the cervical margin and root apical third. The maximum stress of the PDL is reduced when lingual force is applied. From the perspective of PDL stress, the sliding retraction force should be loaded lingually to maintain the force value of 1∼1.5 N.

## Figures and Tables

**Table 1:** Mechanical parameters of the materials.

Material	Young's modulus (MPa)	Poisson'sratio
Tooth	20700	0.3
Periodontal ligament	0.667	0.45
Alveolar bone	14700	0.3
Stainless steel (buccal tube)	20000	0.3

**Table 2:** Force loading and condition setting.

Force loading	Working conditions	
	MBT group	PASS group
Buccal 1 N	Working condition 1	Working condition 2
Buccal 1.5 N	Working condition 3	Working condition 4
Buccal 2 N	Working condition 5	Working condition 6
Lingual 1 N	Working condition 7	Working condition 8
Lingual 1.5 N	Working condition 9	Working condition 10
Lingual 2 N	Working condition 11	Working condition 12
Buccal + lingual 1 N	Working condition 13	Working condition 14
Buccal + lingual 1.5 N	Working condition 15	Working condition 16
Buccal + lingual 2 N	Working condition 17	Working condition 18

**Table 3:** The maximum values of the first principal stresses and the minimum values of the third principal stresses of the PDL and the sites were recorded in all working conditions (unit: MPa).

Loading methods	MBT				PASS			
	Maximum value of first principal stress (MPa)	Site	Minimum value of third principal stress (MPa)	Site	Maximum value of first principal stress (MPa)	Site	Minimum value of third principal stress (MPa)	Site
Buccal 1 N	0.040222	Mesial surface of palatal root apical third	−0.043385	Cervical margin of buccomesial surface	0.039635	Cervical margin of linguodistal surface	−0.043373	Cervical margin of buccomesial surface

Buccal 1.5 N	0.060332	Mesial surface of palatal root apical third	−0.065081	Cervical margin of buccomesial surface	0.059453	Cervical margin of linguodistal surface	−0.06506	Cervical margin of buccomesial surface

Buccal 2 N	0.080445	Mesial surface of palatal root apical third	−0.08677	Cervical margin of buccomesial surface	0.07927	Cervical margin of linguodistal surface	−0.086747	Cervical margin of buccomesial surface

Lingual 1 N	0.037318	Cervical margin of linguodistal surface	−0.034212	Cervical margin of linguomesial surface	0.033873	Cervical margin of linguodistal surface	−0.034805	Cervical margin of buccomesial surface

Lingual 1.5 N	0.055966	Cervical margin of linguodistal surface	−0.051323	Cervical margin of linguomesial surface	0.05081	Cervical margin of linguodistal surface	−0.052208	Cervical margin of buccomesial surface

Lingual 2 N	0.074637	Cervical margin of linguodistal surface	−0.068424	Cervical margin of linguomesial surface	0.067746	Cervical margin of linguodistal surface	−0.069611	Cervical margin of buccomesial surface

Buccal + lingual 1 N	0.036709	Cervical margin of linguodistal surface	−0.030782	Cervical margin of buccomesial surface	0.034406	Cervical margin of linguodistal surface	−0.030069	Cervical margin of buccomesial surface

Buccal + lingual 1.5 N	0.05509	Cervical margin of linguodistal surface	−0.046176	Cervical margin of buccomesial surface	0.051648	Cervical margin of linguodistal surface	−0.045134	Cervical margin of buccomesial surface

Buccal + lingual 2 N	0.073445	Cervical margin of linguodistal surface	−0.061567	Cervical margin of buccomesial surface	0.068813	Cervical margin of linguodistal surface	−0.060138	Cervical margin of buccomesial surface

**Table 4:** Maximum and average von Mises stresses of the PDL (unit: MPa).

Loading methods	MBT			PASS		
	Maximum value (MPa)	Site	Average value (MPa)	Maximum value (MPa)	Site	Average value (MPa)
Buccal 1 N	0.027644	Cervical margin of mesiobuccal surface	0.004233	0.028419	Cervical margin of mesiobuccal surface	0.004325
Buccal 1.5 N	0.041471	Cervical margin of mesiobuccal surface	0.006350	0.042628	Cervical margin of mesiobuccal surface	0.006488
Buccal 2 N	0.055288	Cervical margin of mesiobuccal surface	0.008466	0.056837	Cervical margin of mesiobuccal surface	0.008650
Lingual 1 N	0.018428	Cervical margin of linguodistal surface	0.003202	0.017074	Cervical margin of linguodistal surface	0.003156
Lingual 1.5 N	0.027638	Cervical margin of linguodistal surface	0.004803	0.025612	Cervical margin of linguodistal surface	0.004734
Lingual 2 N	0.036856	Cervical margin of linguodistal surface	0.006405	0.034149	Cervical margin of linguodistal surface	0.006312
Buccal + lingual 1 N	0.020559	Cervical margin of mesiobuccal surface	0.003169	0.020325	Cervical margin of mesiobuccal surface	0.003146
Buccal + lingual 1.5 N	0.030836	Cervical margin of mesiobuccal surface	0.004753	0.030508	Cervical margin of mesiobuccal surface	0.004722
Buccal + lingual 2 N	0.041116	Cervical margin of mesiobuccal surface	0.006338	0.040651	Cervical margin of mesiobuccal surface	0.006291

## Data Availability

The data underlying the findings of the study are available from the corresponding author upon request.
